# Case report of a novel alpha1-antitrypsin null variant in Türkiye: Q0_RİZE_

**DOI:** 10.1186/s12890-024-02900-6

**Published:** 2024-02-21

**Authors:** Dilek Karadoğan, Ünal Şahin, Bettina Dreger, Laura Grandoso, Lourdes Osaba

**Affiliations:** 1 School of Medicine, Department of Chest Diseases, Recep Tayyip Erdoğan University, Rize, Türkiye; 2 Pulmonology Medical Affairs, Grifols Deutschland GmbH, Frankfurt, Germany; 3 Progenika Biopharma, a Grifols company, Derio, Spain

**Keywords:** Emphysema, Alpha1-antitrypsin, Alpha1-antitrypsin deficiency, *SERPINA1*

## Abstract

**Background:**

Alpha1-antitrypsin (AAT) is a serine protease inhibitor that serves as a counterbalance to the activity of elastases, e.g., neutrophil elastase in lung tissue. AAT deficiency (AATD) is a rare disorder usually arising from mutations to the *SERPINA1* gene that codes for AAT. The most common AATD alleles are S and Z which produce ~ 40% and ~ 90% reductions in serum AAT, respectively. Rare genetic variants (> 500 identified) can also be associated with mild to severe AATD.

**Results:**

This report describes a novel mutation of *SERPINA1* producing AATD, which we have designated, Q0_RİZE_. This mutation was identified in a 44-year-old woman admitted with massive hemoptysis and treated with bronchial artery embolization. Computed tomography revealed centriacinar and panacinar emphysema with prominent air entrapment, atelectasis, and localized bronchiectasis. Serum AAT was < 0.27 g/L (below detection limit). Genetic analysis showed homozygous deletion of exons I to III.

**Conclusions:**

Although many *SERPINA1* variants have been identified, variants with large deletions and identified in a homozygous individual, as seen in this case with Q0_RIZE,_ are uncommon. AATD is an underdiagnosed and undertreated disease. Wider screening of COPD patients could result in earlier diagnosis and treatment that could preserve lung function.

## Background

Under normal physiologic conditions, alpha1-antitrypsin (AAT), a serine protease inhibitor, acts to counteract the activity of elastases, primarily neutrophil elastase (NE) in the lungs. AAT is a serum anti-protease synthesized primarily in the liver and transported to the lungs through the bloodstream. When AAT levels are deficient (AAT deficiency (AATD)) the regulation of NE activity is disrupted, and NE activity is increased potentially leading to destruction of lung tissue and impaired gas exchange [1]. 

AATD is a relatively rare inherited disorder that increases the propensity for lung and liver disease. The *SERPINA1* gene codes for the AAT protein. Genetic analyses have revealed that the M-allele of this gene is associated with normal serum levels of AAT and is the most common allele. At present, the pathogenic alleles most often associated with reduced serum AAT (90% of AATD cases) are the S- (40% reduction in serum AAT) and Z-alleles (90% reduction in serum AAT) [2, 3]. More than 500 additional variants of the *SERPINA1* gene have been identified [4, 5]. Approximately 40% of these variants are associated with some degree of AATD [6] and may be responsible for up to 17% of clinical cases of AATD [7, 8]. 

Despite the large amount of research identifying *SERPINA1* variants and the clear clinical significance early diagnosis and treatment, AATD is underdiagnosed and consequently undertreated. This case report summarizes the clinical findings for a patient with a previously undescribed *SERPINA1* genetic variant.

### Case presentation

**Fig. 1:**
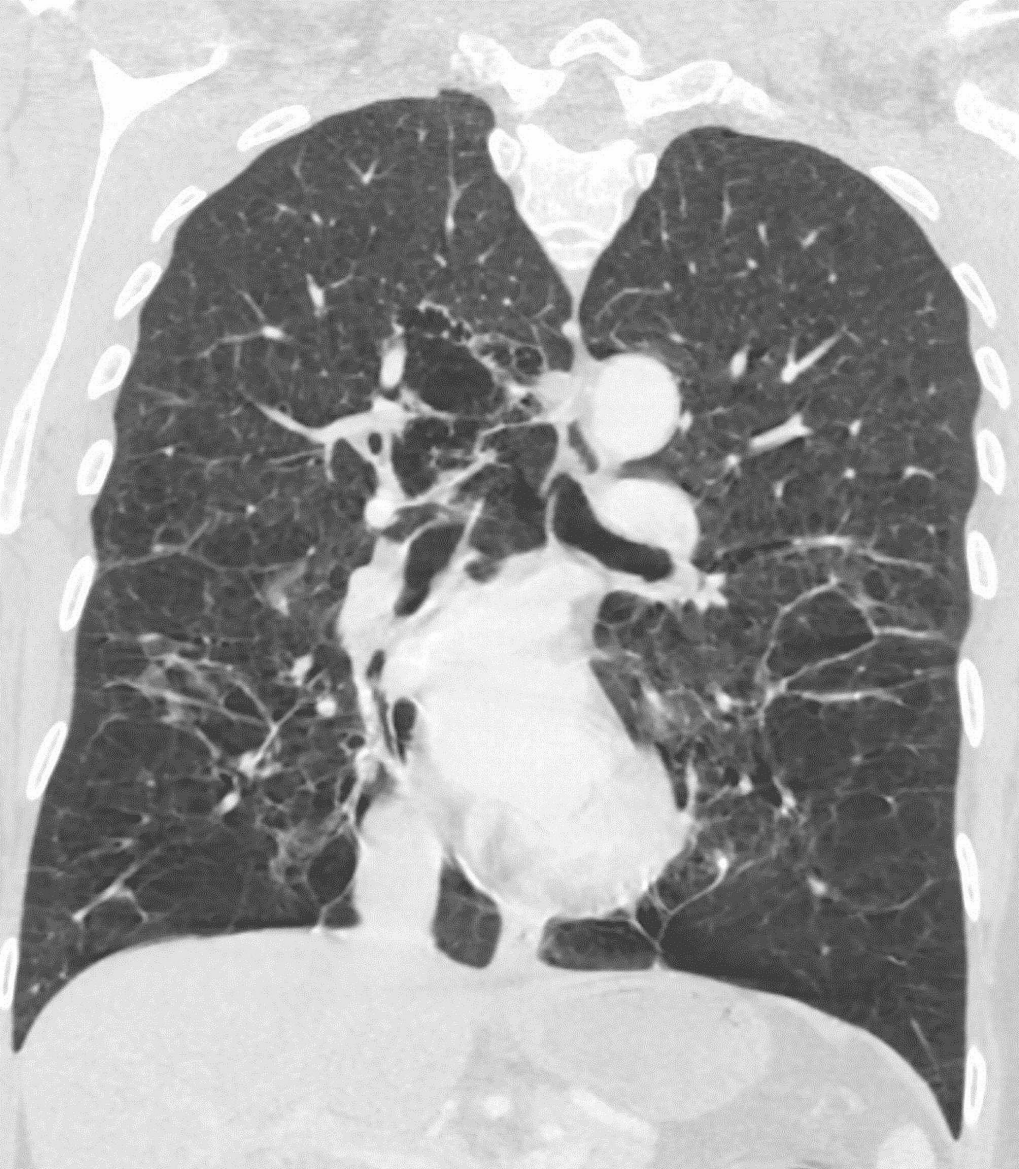
Coronal image of the thorax CAT scan. CAT scan of a 44-year-old female patient with alpha-1 antitrypsin deficiency (AATD) due to a novel alpha1-antitrypsin null variant, Q0_RİZE_, showing widespread centriasinar and panasinar emphysema predominantly in the lower lobes

**Fig. 2:**
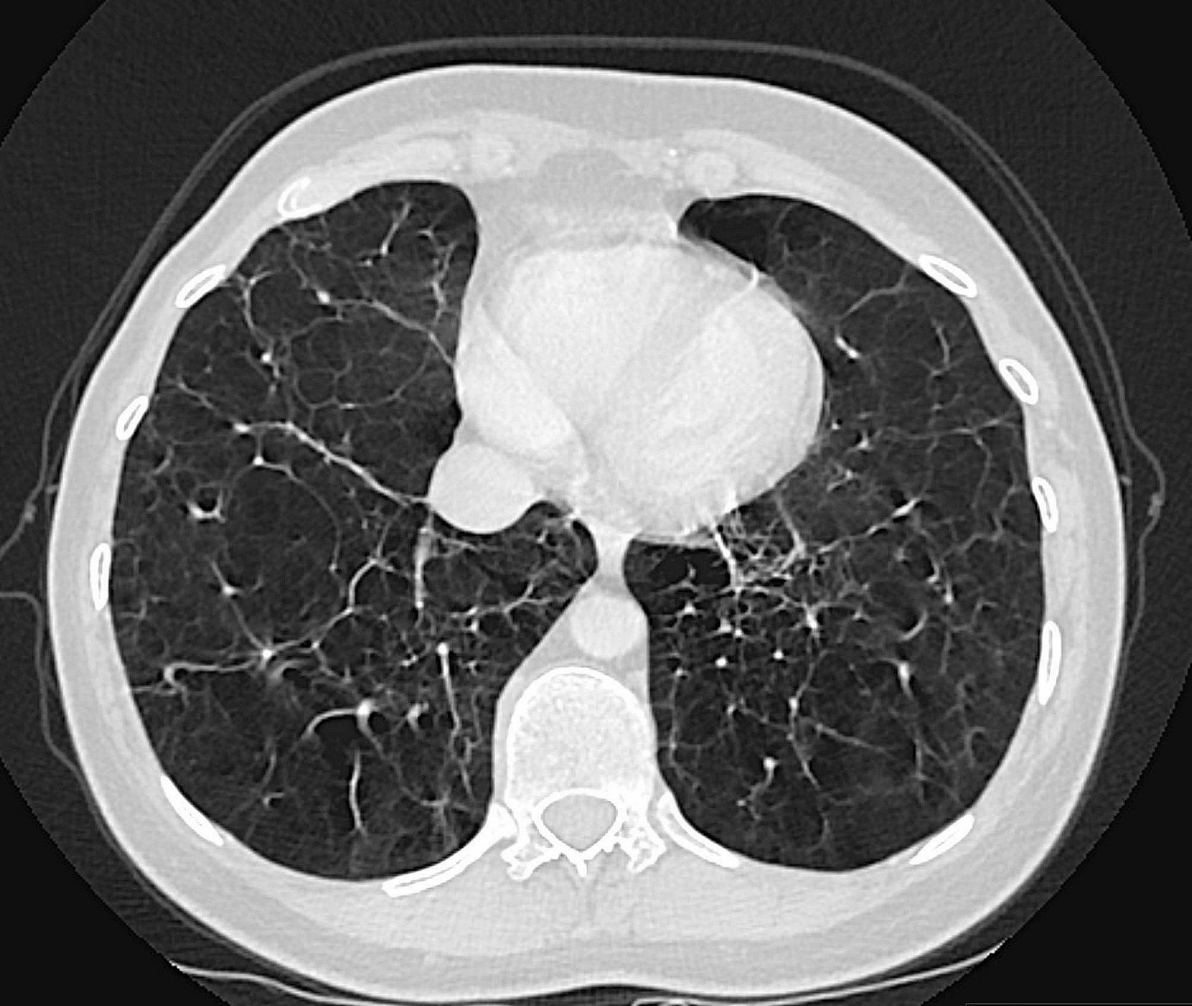
Transverse image of the thorax CAT scan. CAT scan of a 44-year-old female patient with alpha-1 antitrypsin deficiency (AATD) due to a novel alpha1-antitrypsin null variant, Q0_RİZE,_ showing widespread centriasinar and panasinar emphysema predominantly in the lower lobes

**Fig. 3:**
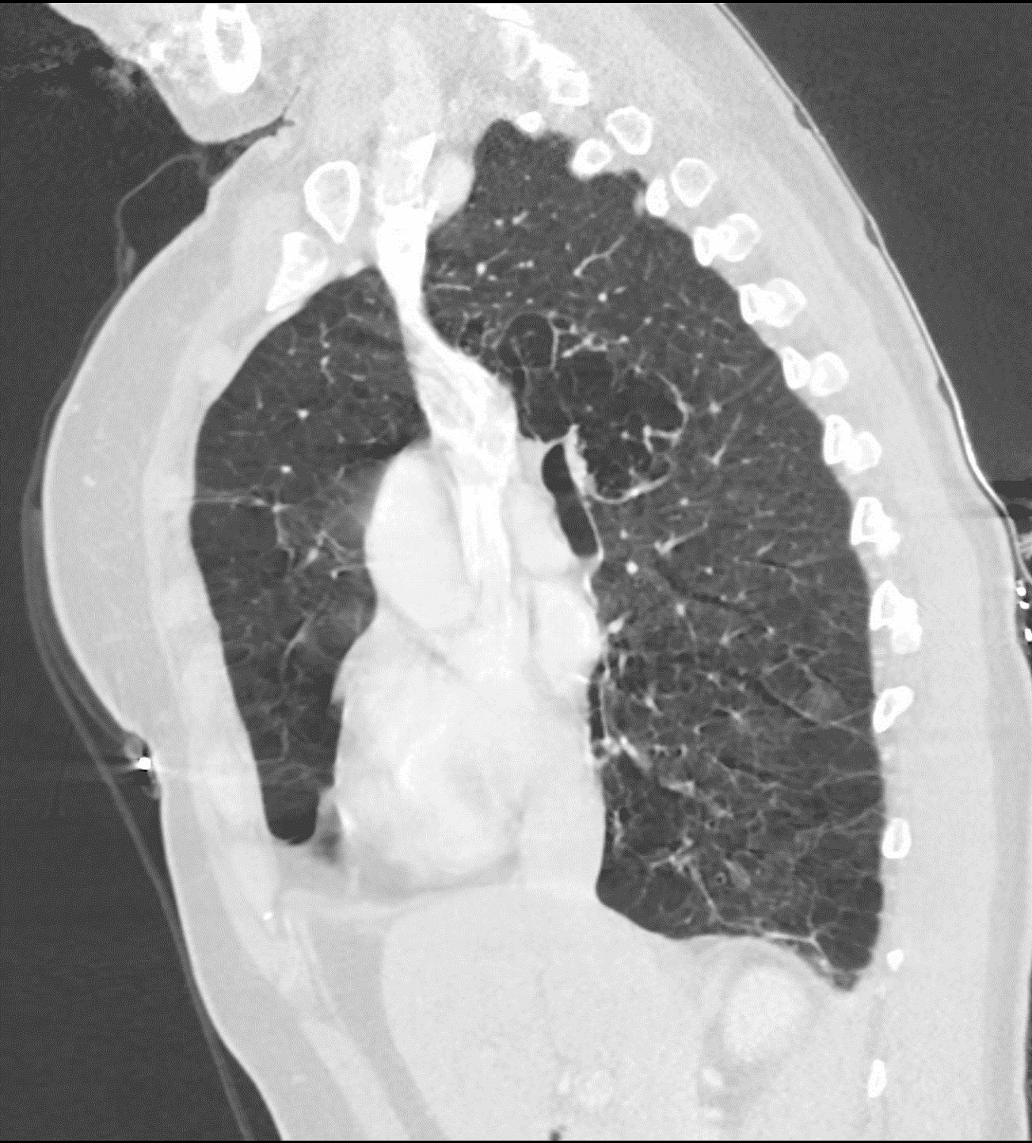
Sagittal section of thorax CAT scan. CAT scan of 44-year-old female patient with alpha-1 antitrypsin deficiency due to a novel alpha1-antitrypsin null variant, Q0_RİZE,_ showing an area of cystic bronchiectasis in the central area of the upper lobe posterior segment of the right lung

This 44-year-old female patient was hospitalized in the pulmonary clinic due to massive hemoptysis in September 2022. The interventional radiology department was consulted, and embolization of her hypertrophied bronchial arteries was performed the same day. Chest computed axial tomography (CAT) scans were performed and widespread emphysema was detected. The CAT scan showed centriacinar and panacinar emphysematous air entrapment in both lungs - more prominent in the lower lobes (Figs. 1 and 2). Atelectasis and volume loss were observed in the right middle and lower lobes and in the left lingular segment. Localized tractional bronchiectasis was seen in the right middle lobe, lingula inferior and both lower lobes. In the posterior segment of the right upper lobe, an area of cystic bronchiectasis was seen that appeared to merge with an area in the central lobe (Fig. 3).

For medical history, the patient reported fatigue and dyspnea with exercise since her teenage years and was diagnosed with asthma at that time. Her father is the only first degree relative with confirmed chronic obstructive pulmonary disease (COPD). She also has a fraternal cousin (male) that was diagnosed to be homozygous ZZ and is on AAT augmentation therapy.

The patient is currently employed and reports occupational exposure to dust in her job at a local tea factory. She reported that she is a former smoker (starting at 18 years old) with a 10 pack-year exposure prior to quitting in 2014. She also reported that her husband is a heavy smoker and therefore she has ongoing secondhand and thirdhand exposure to cigarette smoke.

The patient has seen an increase in the severity of her pulmonary symptoms over the past three years. Her recent symptoms (May 2023) include dyspnea (primarily with exertion) and fatigue. Her dyspnea score on the Modified Medical Research Council (mMRC) scale was 2 on a 0–4 scale [9–11]. Her Global Initiative for Chronic Obstructive Lung Disease (GOLD) COPD status was determined to be Stage B [12]. Her oxygen saturation was 98% and lung auscultation was normal. Her pulmonary function tests showed forced expiratory volume in one second (FEV1) of 1.11 L (37% of predicted value), forced vital capacity (FVC) of 2.23 L (64% of predicted) and FEV1/FVC ratio of 50%. Her liver enzymes were within the normal range (alanine transaminase (ALT) 9 U/L (reference range 0–35 U/L and aspartate transaminase (AST) 12 U/L (reference range 0–35 U/L).

**Fig. 4:**
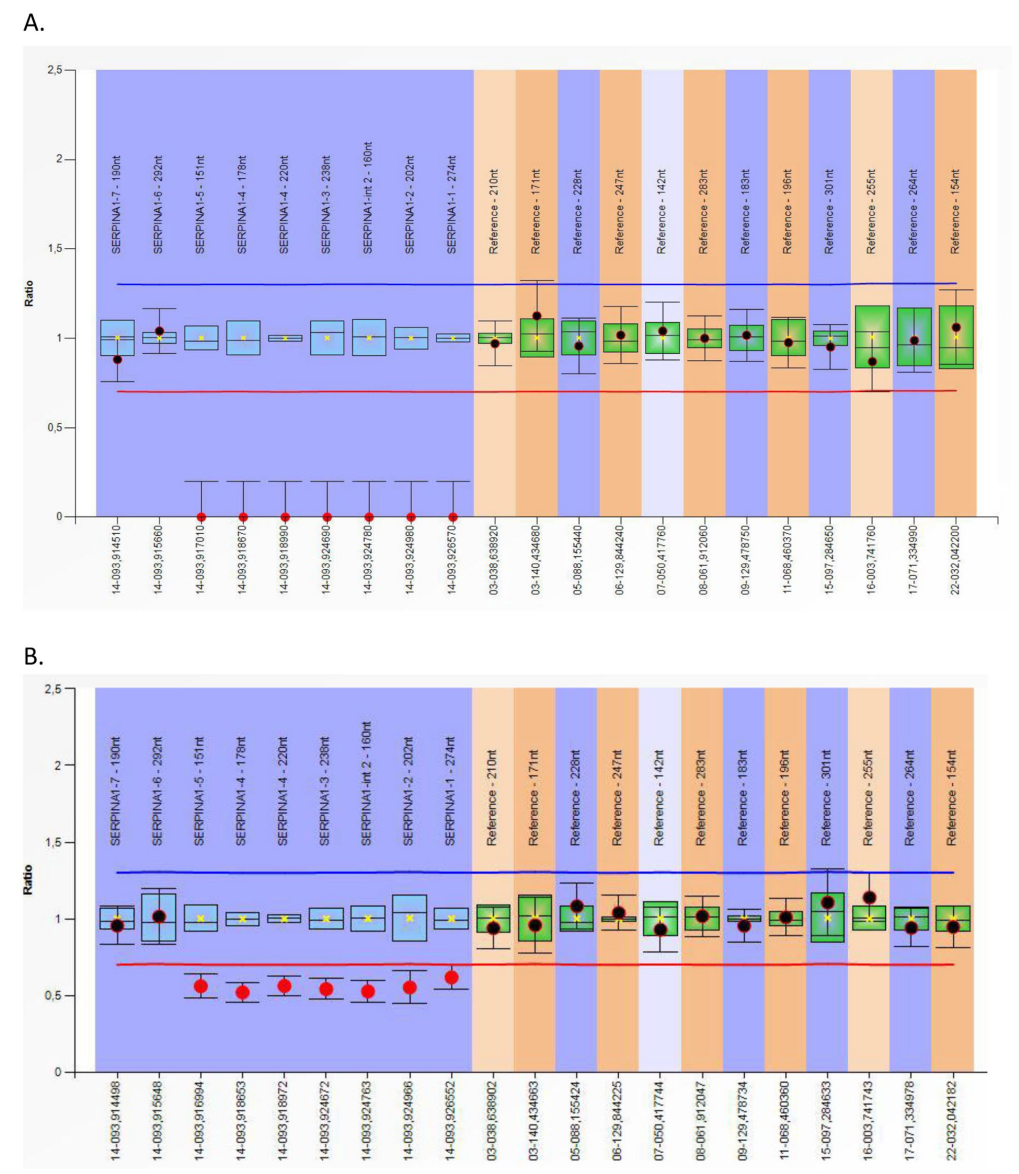
MLPA results for the index patient (**A**) and a relative (**B**). The *SERPINA1* exon numbering used in this P459-A2 *SERPINA1* product is the exon numbering from the RefSeq transcript NM_001127701.1 (transcript variant 5), so that non-coding exon (Ia, Ib and Ic) are numbering as 1, 2 and 3, respectively, and exon II, III, IV and V are indicated as 4, 5, 6 and 7, respectively. Calculated probe ratios normalized to the reference samples are indicated on the y-axis. Arranging probes by chromosomal location reveals the homozygous deletion (probe ratio 0) in the patient’s sample (**A**) and heterozygous deletion (probe ratio around 0.5) in the relative’s sample (**B**)

The patient’s serum AAT level was < 0.27 g/L, below the limit of detection (Goldsite Diagnostics, Inc., Shenzhen, China; normal values 0.9-2.0 g/L). A dried blood sample (DBS) was obtained and sent to Progenika Biopharma for genetic analysis (see Methods for details). Sequencing results showed normal coverage for amplicons corresponding to exons IV and V, but extremely low coverage (similar to the negative control) for all other amplicons. This could be interpreted as a homozygous deletion of exons I to III. This was confirmed by multiplex ligation-dependent probe amplification (MLPA) (Fig. 4). Using an exon-based description, this variant is genetically described as c.(?_-573)_(917 + 1_918-1)del. Large deletions are extremely infrequent in *SERPINA1* and this is the first description of this variant.

The patient is currently being treated with a long-acting beta agonist, formoterol, and a long-acting muscarinic antagonist, tiotropium. These medications are supplemented with a short-acting beta agonist, salbutamol, and a short-acting muscarinic antagonist, ipratropium bromide, as needed. AAT replacement therapy was initiated in this patient but was discontinued after the second dose due to an allergic skin reaction. The serum IgA level on this patient was normal (1.81 g/L).

**Fig. 5:**
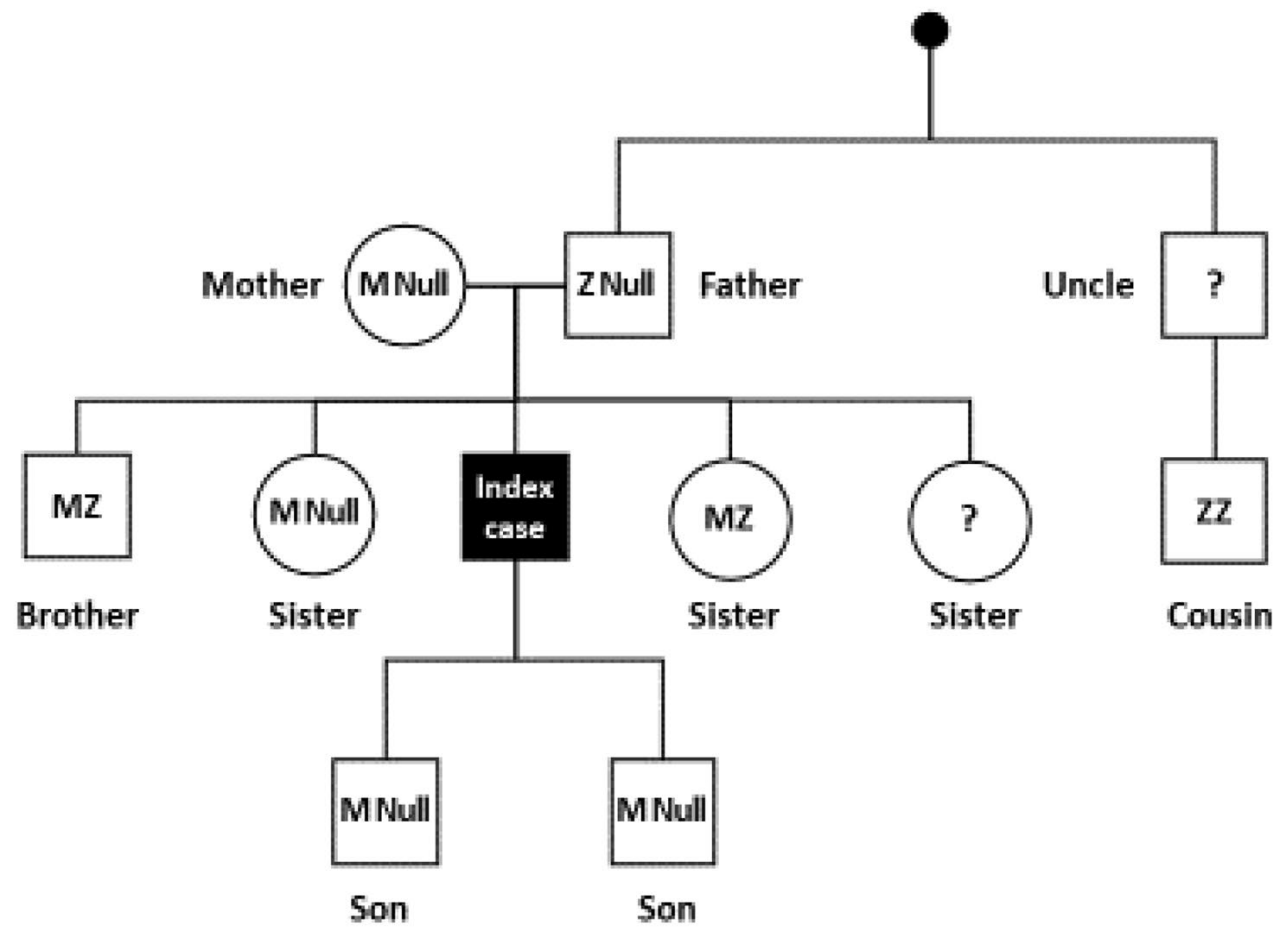
**Family tree for the patient (index case) and close relatives.** Genetic analysis results are shown for first- and second-degree relatives of the patient whose case is reported here

DBS samples were obtained from several members of the patient’s family for genetic screening. *SERPINA1* gene sequencing and MLPA analysis were performed for these relatives. The results are shown in the family tree in Fig. 5. The patient presented here (index case) is homozygous for the Q0_RIZE_ variant having received a copy of the null variant from both parents. Her father found to be PiZ-Q0_RIZE_ is her only first degree relative diagnosed with COPD. Her cousin has been found to be homozygous ZZ and is on AAT augmentation therapy. Her sisters, brother and sons all have the normal M allele in combination with the Z or null allele and have not been diagnosed with lung or liver disease.

## Methods

DBS were obtained from the patient and family members and sent to Progenika for analysis. The first analysis was performed using the A1AT genotyping Test (Progenika Biopharma, SA, Derio, Spain) on the Luminex 200 (Luminex, Austin, TX, USA). This multiplex PCR (polymerase chain reaction) and hybridization test allows the simultaneous identification of 14 genetic variants, corresponding to the most frequent alleles found in AATD patients. If inconclusive, results were obtained with the A1AT Genotyping Test, full sequencing of the *SERPINA1* gene was performed using Next Generation Sequencing. The amplicon library was prepared covering the full gene (three non-coding and four coding exons) and sequenced on MiSeq (Illumina, Inc, San Diego, CA, USA). Results were confirmed by multiplex ligation-dependent probe amplification (MLPA) analysis using SALSA MLPA P459-A2 *SERPINA1* kit (MRC Holland, Amsterdam, Netherlands). MLPA is based on the amplification of a set of probes that each detect a specific DNA sequence of approximately 60 nucleotides in length. The SALSA MLPA Probemix P459-A2 *SERPINA1* contains 21 MLPA probes: nine probes for the *SERPINA1* gene and 12 reference probes that detect autosomal chromosomal locations. MLPA is a relative technique: only relative differences can be detected by comparing the MLPA peak patterns of DNA samples. The relative intensity of each individual probe, as compared to the relative probe intensity in various reference DNA samples, reflects the relative copy number of the corresponding target sequence in a sample. A deletion of one or more target sequences is visible as a relative decrease in intensity.

## Discussion

In this article, we report the case of a patient with severe emphysema and AATD. Genetic analysis for this patient showed a homozygous deletion of exons I to III, a variant we are calling Q0_RIZE_. Large deletions such as this are uncommon as null mutations are most commonly caused by single base deletions [5]. However, the null mutations Q0_isola de procida_ [13] and Q0_riedenburg_ [14] were found to be due to deletions of exons II to V. It is even more unusual that this patient appears to be homozygous for this allele.

In this case, a novel null variant was identified in a homozygous patient. This patient had serum AAT levels below the limit of detection and significant emphysema likely complicated by a 10 pack-year history of smoking and on-the-job exposure to dust. AATD was identified in this patient at 44 years of age and with advanced disease. The patient is currently being treated with inhaled dual bronchodilators and with short acting bronchodilators as needed.

AATD is an underdiagnosed disease due to the lack of widespread testing. Due to the lack of a diagnosis in affected individuals, it is consequently undertreated. Earlier detection of AATD could lead to earlier treatment, including assistance with reduction of environmental hazards (e.g., smoking cessation therapy for the patient and household members and workplace risk reduction) and AAT augmentation therapy (if feasible and warranted), which could help improve or maintain the patient’s lung function and quality of life.

As previously noted, a large number of variants of *SERPINA1* have been identified. The clinical characteristics of some of these variants have not fully described. When previously undescribed *SERPINA1* variants are tentatively identified, full characterization of the patient’s medical history, lab values and genetic profile could be helpful in expanding the knowledge base of AATD and in providing guidance for selecting the best course of treatment.

## Conclusions

- We report a patient with severe emphysema hospitalized for massive hemoptysis. Genetic analysis revealed the patient was homozygous for a previously undescribed *SERPINA1* variant: Q0_RIZE_.
- This patient had a delayed diagnosis due to inadequate access to advanced diagnostic technology and genetic analysis.
- Reporting this and other novel mutations and their clinical profiles is important to advance our understanding of AATD and improving its diagnosis and treatment.

## Data Availability

The data that support the findings of this study are not openly available due to reasons of confidentiality and are available from the corresponding author upon reasonable request. Data are located in controlled access data storage at Recep Tayyip Erdoğan University, School of Medicine, Department of Chest Diseases.

## Abbreviations

**AAT**: Alpha1-antitrypsin
AATD: Alpha1-antitrypsin deficiency
ALT: Alanine transaminase
AST: Aspartate transaminase
CAT: Computed axial tomography
COPD: Chronic obstructive pulmonary disease
FEV1: Forced expiratory volume in one second
FVC: Forced vital capacity
GOLD: Global Initiative for Chronic Obstructive Lung Disease (GOLD) COPD status
mMRC: Modified Medical Research Council dyspnea score
NE: Neutrophil elastase
